# AAV viral vectors as therapeutic interventions for inherited or non-inherited cardiac disorders: current aspects and future prospects

**DOI:** 10.3389/fmed.2025.1678325

**Published:** 2025-10-16

**Authors:** Vivek Kumar, Kalimuthu Kalishwaralal, Charu Chauhan, Gurpal Singh, Ravi Pratap Barnwal, Sachin Sharma

**Affiliations:** ^1^Michigan Neuroscience Institute, University of Michigan, Ann Arbor, MI, United States; ^2^Division of Cancer Research, Interactive Research School of Health Affairs, Bharati Vidyapeeth, Pune, India; ^3^Department of Biosciences, Graphic Era University, Dehradun, India; ^4^University Institute of Pharmaceutical Sciences, Panjab University, Chandigarh, India; ^5^Department of Biophysics, Panjab University, Chandigarh, India

**Keywords:** tachyarrhythmia, hypertrophic cardiomyopathy, ACE inhibitors, β-blockers, EV-encapsulated AAVs

## Abstract

Ischemic and non-ischemic cardiac diseases including arrhythmogenic cardiomyopathy and myocardial infarction, remain one of the leading causes of death worldwide despite significant advances in cardiovascular therapeutics. Current treatment strategies such as β-blockers, angiotensin-converting-enzyme inhibitors, and cardiac surgical interventions that include implantations of pacemakers and cardioverter-defibrillators are effective but often associated with serious side effects. In recent years, multiple cell-based therapies have emerged, aiming either to regeneration of myocardial or myofascial tissues or to correct defective gene using gene-transfer tools. Adeno-associated virus (AAVs), initially identified as contaminants of adeno-virus preparations, have since become one of the most important viral vectors for gene-transfer, especially in mammalian cells. This review analyzes and summarizes various AAV serotypes utilized in gene therapy programs for preclinical and clinical assays for cardiac disease.

## Introduction

**Figure 1:**
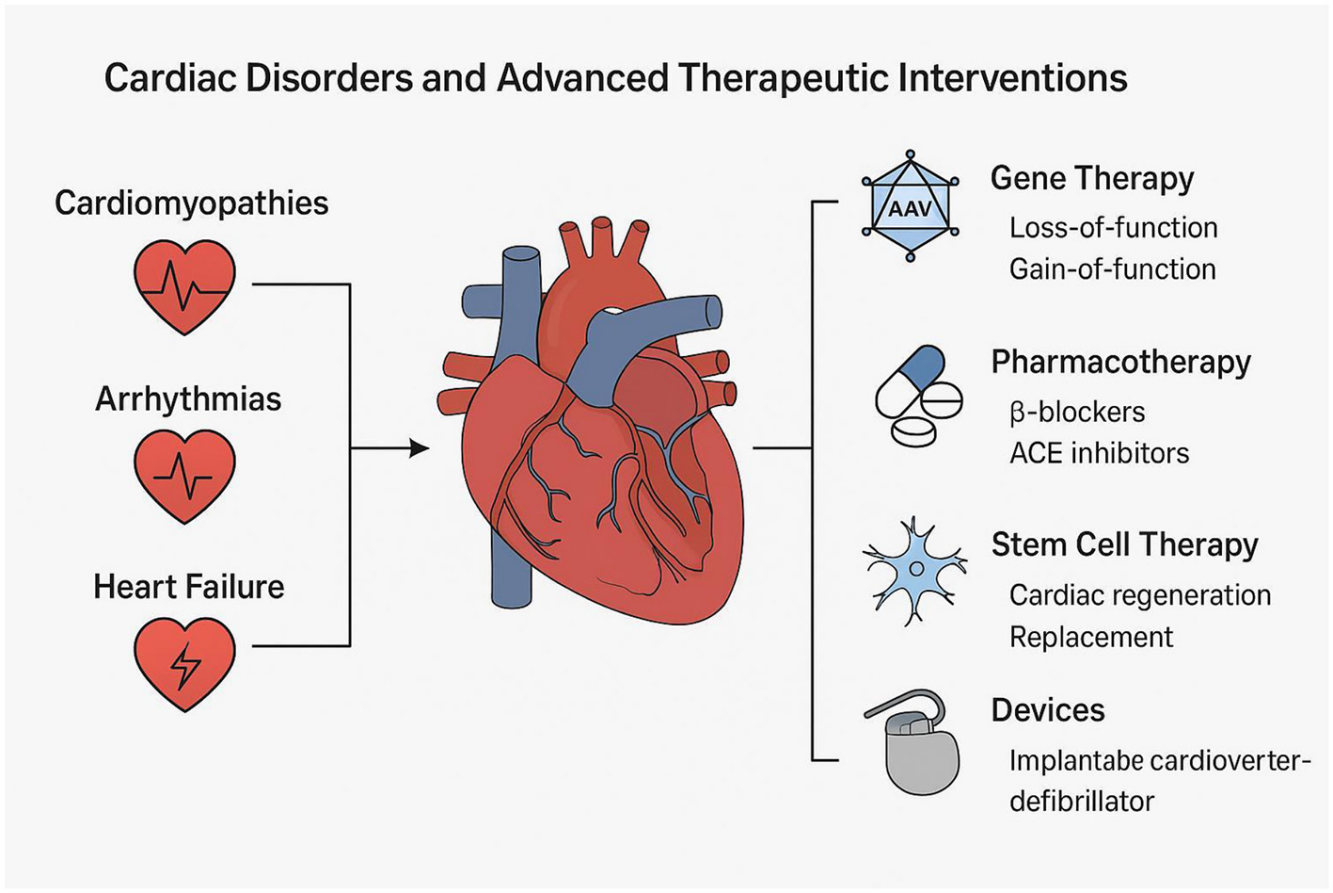
A diagram that illustrates various therapies under consideration for cardiac disorders.

Inherited and acquired cardiac diseases, such as arrhythmogenic cardiomyopathy and myocardial infarction (MI), remain leading contributors to heart failure and cardiovascular mortality globally (1). Despite the currently available pharmacological agents like β-blockers and ACE inhibitors, and device-based interventions such as pacemakers and defibrillators, the long-term management of these conditions still remains suboptimal for many patients due to limited efficacy, adverse effects, or progression to end-stage heart failure necessitating urgent need for alternative therapeutic agents (2) (Figure 1).

Arrhythmias, particularly atrial fibrillation, further complicate clinical outcomes and affect over 40 million individuals worldwide, highlighting an urgent need for more precise and durable treatment strategies (3). In this context, gene therapy offers the potential for long-lasting correction of molecular dysfunctions at the cellular level. Among available gene delivery tools, adeno-associated viruses (AAVs) have emerged as leading candidates due to their relatively low immunogenicity, cardiac tissue tropism, and ability to mediate long-term gene expression in non-dividing cells (4–6). Recent advancements in AAV engineering, including capsid design and promoter specificity, have expanded their utility for cardiovascular applications. Several serotypes have been evaluated in preclinical and clinical studies for their ability to restore cardiac function through gain-of-function or loss-of-function strategies targeting ion channels, contractility proteins, or regulatory kinases (7, 8). However, despite encouraging results in animal models, clinical translation has faced setbacks due to challenges such as immune clearance, inadequate delivery, and scalability issues.

Cardiac diseases can broadly be categorized as inherited genetic disorders, such as hypertrophic cardiomyopathy (HCM), dilated cardiomyopathy (DCM), and arrhythmogenic right ventricular cardiomyopathy (ARVC), and non-inherited (acquired) conditions, such as myocardial infarction (MI), ischemic cardiomyopathy, and pressure-overload–induced heart failure. These two categories differ not only in etiology but also in AAV vector selection, delivery strategy, and therapeutic timing. This review provides a detailed overview of various AAV serotypes and their use in cardiac gene therapy programs, highlighting their biological properties, preclinical performance, and clinical trial outcomes.

## AAV serotypes in gene-therapies

AAV1, originally isolated from human tissues, shows tissue-tropism for heart, skeletal muscles, and the central nervous system (CNS). AAV1 serotype primarily uses sialic acid as a surface receptor for binding on cell surface (9, 10). Notably, recombinant AAV1 (rAAV1) was the first viral vector approved for gene therapy (11). Glybera, an AAV1-based drug, was approved in the market for the treatment of a rare monogenic genetic disorder, lipoprotein lipase deficiency (12, 13). Alipogene tiparvovec, using AAV1 carrying the LPL^s447x^ gene variant, was tested in clinical trials for the same condition (14).

AAV2 is a non-pathogenic, single-stranded (ss) DNA vector (~4.7kb) (15). It can transduce many cell-types except MB-02 human megakaryocytic leukemia cells (16). AAV2 binds to heparan sulfate proteoglycans (HSPG) as primary receptor, with human fibroblast growth factor receptor 1 (FGFR1), human hepatocytes growth factor receptor (hHGFR), laminin receptor (LR), αVβ5, and α5β1 integrins as co-receptors. AAV2 was first used clinically in Canavan disease to deliver Aspartoacylase gene (ASPA) (17), and later in Parkinson's disease trials to deliver neurturin (18).

AAV3, also isolated from humans, shares receptor usage with AAV2 (HSPG, FGFR1, hHGFR, LR) and is increasingly used due to its higher transduction efficiency in both *in-vitro* and *in-vivo* settings. It uses hHGFR as lateral coreceptor in human cancerous cells and non-human primates (NHP) hepatocytes. In hemophilia-B clinical trials, AAV3 successfully transduced primary human hepatocytes and humanized mice using liver-specific hFIX promoters (19). AAV4, identified in NHPs, mainly from green monkeys, lacks heparin-binding specificity (20). Its capsid undergoes post-translational modifications such as ubiquitination (21). AAV4 shows transduction preference for ependymal cells, and lung and heart tissues in murine model (22). AAV5, one of the most genetically divergent serotypes, uses sialic acid as a primary receptor and platelet-derived growth factor (PDGFR) α and β as coreceptors. It is highly effective in transducing murine photoreceptor cells. AAV5-delivered valoctocogene roxaparvovec, which encodes human B domain-deleted FVIII (hFVIII-SQ), is being tested in phase1/2 clinical trials for hemophilia A (23, 24). AAV6 shares serological profiles with AAV1 and binds to sialylated proteoglycans and heparan sulphate, using epidermal growth factor receptor (EGFR) as a co-receptor. It transduces various tissues, especially liver and skeletal muscles in mice and dogs. AAV6-mediated delivery of βARKct, a peptide inhibitor targeting GRK2, has preserved β-adrenergic responsiveness in post-MI heart failure models (25).

AAV7, a serotype isolated from Rhesus macaques, does not bind heparin or other glycans. Its capsid undergoes phosphorylation, acetylation, and glycosylation. AAV7 effectively transduces murine neurons, photoreceptors, and hepatocytes in both murine and human tissues. Its low seroprevalence in humans makes it attractive for clinical translation (26, 27). AAV8, also isolated from Rhesus macaque monkeys in 2002, uses LR as a primary receptor (similar to AAV2 and AAV3). It is purified using dual ion-exchange chromatography or iodixanol gradient centrifugation (28). AAV8 demonstrates strong liver tropism and high transduction efficiency both skeletal and cardiac muscles (28, 29). AAV9, identified and isolated in 2004, uses terminal N-linked galactose along with LR as coreceptors, with additional affinity for putative integrins. It is highly efficient in transducing a wide range of tissues and is uniquely capable of crossing the blood-brain barrier to infect neurons, astrocytes, and glial cells (30, 31). It can also penetrate endothelial barriers to reach cardiac muscle (32). Detailed information for these AAV serotypes and their preclinical and clinical usage are provided in Table 1.

**Table 1:** The current table provides detailed information about several AAV serotypes and their associated preclinical and clinical studies.

**AAV Serotype**	**Origin**	**Primary receptors**	**Tissue tropism**	**Gene therapy applications**	**Delivery method**	**Stage**	**Key references**
AAV1	Human	Sialic acid	Heart, skeletal muscle, CNS	*SERCA2a* for heart failure (CUPID trial); LPL deficiency (Glybera)	Intracoronary, intramuscular	Preclinical and Clinical	Scott et al. (87), Greenberg et al. (88), Ross et al. (89)
AAV2	Human	HSPG, FGFR1, hHGFR, Laminin R	Liver, CNS, retina	Canavan disease (*ASPA*), Parkinson's disease (*neurturin*)	Intracranial, systemic	Clinical	Leone et al. (90); Marks et al. (18)
AAV3	Human	HSPG + co-receptors (FGFR1, hHGFR, LR)	Liver, hepatocytes	*FIX* for hemophilia B	Intravenous	Preclinical	Brown et al. (19)
AAV4	NHP (African green monkey)	Unknown (not heparin binding)	Ependymal cells, heart, Retinal cells	Neural gene transfer in rodents	Intrathecal, intraventricular	Preclinical	Le Meur et al. (91)
AAV5	Human	Sialic acid, PDGFR α/β	Retina, CNS, photoreceptors	*hFVIII-SQ* for hemophilia A (valoctocogene roxaparvovec)	Intravenous, subretinal	Phase 1/2	Rangarajan et al. (92, 93)
AAV6	Hybrid of AAV1/AAV2	Sialylated proteoglycans, HSPG, EGFR	Skeletal & cardiac muscle	*βARKct* for GRK2 inhibition in post-MI heart failure	Retrograde venous injection	Preclinical	Raake et al. (25)
AAV7	NHP (Rhesus monkey)	Not heparin binding	Hepatocytes, retina, neurons	Liver-directed gene therapy	Intravenous	Preclinical	Shao et al. (94)
AAV8	NHP	Laminin R, integrins	Liver, skeletal muscle, cardiac	Liver gene therapy	Intravenous	Preclinical	Wang et al. (95); Fang et al. (96)
AAV9	Human/NHP	N-linked galactose, integrins, Laminin R	Heart, CNS, skeletal muscle	*MYBPC3, S100A1, cBIN1* for cardiomyopathy, heart failure; crosses BBB	Systemic, intramyocardial	Preclinical	Chen et al. (56); Greer-Short et al. (38)
AAVrh.74	NHP (Rhesus monkey)	Similar to AAV8/9	Cardiac, skeletal muscle	*PKP2a* in ARVC models	Systemic	Preclinical	van Opbergen et al. (35)
AAV2i8	Engineered hybrid	Modified for enhanced tropism	Cardiomyocytes	Heart failure therapy (NCT04179643)	Intracoronary	Phase 1	ClinicalTrials.gov (NCT04179643) (97)

## Implications of AAVs in inherited and acquired cardiac diseases

**Figure 2:**
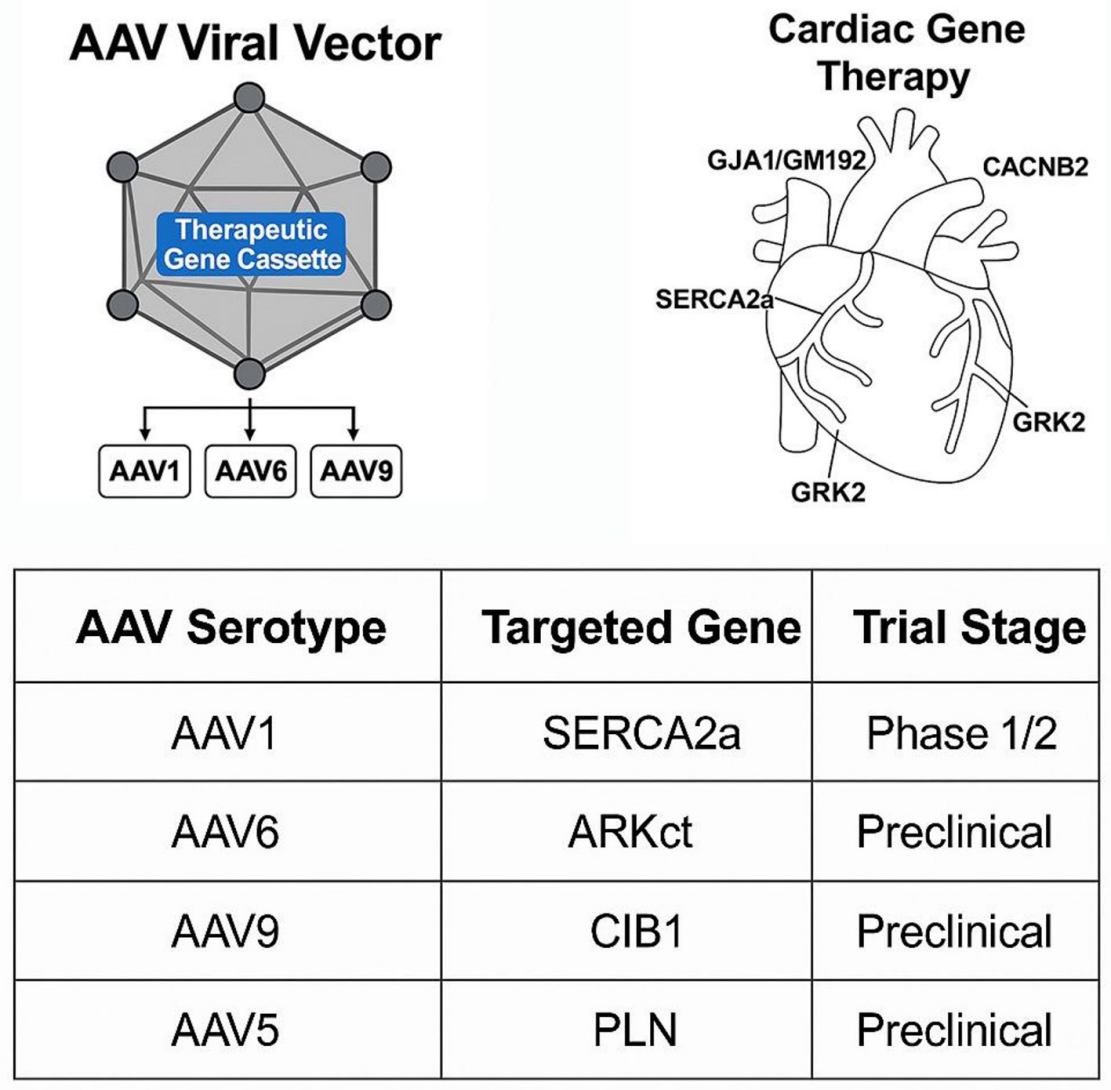
AAV serotypes in cardiac clinical trials and gene therapy applications. This figure summarizes the landscape of AAV serotypes utilized in cardiac gene therapy trials. The schematic illustrates target organs and serotype-specific delivery pathways, with emphasis on AAV1 (CUPID trial), AAV9 (preclinical success), and engineered variants. The table below the schematic lists clinical trials involving these serotypes, their therapeutic targets (e.g., SERCA2a, MYBPC3), and outcomes. Clinical-stage trials are marked, highlighting translational challenges and current limitations.

Cardiac gene therapy holds significant promises for both inherited and acquired cardiac diseases, such as arrhythmogenic cardiomyopathy (ACM) and MI. Currently, treatment options such as heart transplantation and endovascular procedures are available for advanced cases. However, for most cardiac conditions, the existing symptomatic therapies remain insufficient and yield limited success. In this section, based on current understanding, we discuss therapeutic applications of AAV in cardiac gene therapy (Figure 2, Table 2).

**Table 2:** Summary of AAV applications in inherited vs. acquired cardiac diseases.

**Disease TYPE**	**EXAMPLE**	**Gene TARGET**	**AAV SEROTYPE**	**MODEL**	**OUTCOME**
INHERITED	HCM (*MYBPC3*)	MYBPC3	AAV9	Mouse	Hypertrophy reversed (38)
INHERITED	ARVC	PKP2a	AAVrh.74	Mouse	Arrhythmia reduced (35)
INHERITED	Barth Syndrome	TAZ	AAV9	KO mice	Lethality and fibrosis rescued (36)
ACQUIRED	MI	βARKct	AAV6	Pig	Contractility improved (25)
ACQUIRED	Chronic HF	cBIN1	AAV9	Dog	↑ LVEF, ↓ remodeling (42)
ACQUIRED	General HF	SERCA2a	AAV1	Human	CUPID2 failed to meet endpoint (44)

*Preclinical studies:* adeno-associated virus (AAV)-based gene therapies have shown considerable promises across a wide range of cardiovascular diseases. These disorders can be broadly classified into inherited (genetically driven) and non-inherited or acquired (typically secondary to ischemic, metabolic, or pressure-related injury). While both categories can benefit from gene modulation strategies, the therapeutic targets, vector choice, and delivery mechanisms often differ. This section explores the specific serotype-gene combinations that have shown efficacy in preclinical or clinical models for each category.

A) ***Inherited cardiomyopathies***. These are typically monogenic diseases caused by pathogenic mutations in structural or regulatory genes critical for cardiomyocyte function. Since these disorders are often manifest early and follow well-defined molecular pathways, they are the attractive targets for gene replacement or correction via AAV viral vectors. We include some of these examples below in sub-sections.

Arrhythmogenic cardiomyopathy is a cardiac muscle disorder characterized by arrhythmias and associated with ischemia, hypertension, or valvular heart disease (33). The characteristic pathological feature of ACM is the replacement of ventricular myocardial tissues with fibrofatty tissues. T-box 5 (TBX5) is a key transcription factor involved in cardiac development, has been shown to play a crucial role—loss of ventricular TBX5 causes cardiac dysfunction and sudden death due to arrhythmias. Systemic delivery of AAV9-TBX5 in knockout mice normalized TBX5 protein level and re-established TBX5-dependent transcriptome, resulting in reduced arrhythmia and improved cardiac function (34).

In another preclinical study, loss of plakophilin-2 function, which causes arrhythmogenic right ventricular cardiomyopathy (ARVC), was corrected through delivery of PKP2a using a recombinant AAVrh.74 vector. This intervention ameliorated symptoms of both early and advanced disease stages in PKP2-cKO mice (35). Similarly, AAV-mediated gene replacement has shown therapeutic potential in Barth syndrome, an X-linked cardiomyopathy caused by mutations in the Tafazzin (Taz) gene. Taz knockout mice exhibited postnatal lethality due to cardiac failure. Neonatal administration of AAV-hTaz rescued lethality and improved cardiac dysfunction and fibrosis in both global and cardiomyocyte-specific Taz-KO mice using an AAV9 vector driven by the CMV promoter (36).

Hypertrophic cardiomyopathy (HC), affecting approximately 0.2% to 0.5% of global adult population, is often associated by loss-of-function mutations in myosin binding protein C3 (MYBPC3) (37). In a symptomatic MYBPC3-deficient mouse model, restoring cardiac MYBPC3 expression level using AAV9 and cis-regulatory element TN-201 reversed hypertrophy, improved systolic and diastolic functions, and prolonged survival (38). These findings suggest that both AAV9 and AAVrh.74 effectively mediate in genetically defined cardiomyopathies through systemic or cardiac-specific gene delivery in preclinical settings.

B) ***Acquired cardiac diseases***. Diseases such as myocardial infarction (MI), ischemic cardiomyopathy, and chronic heart failure (HF) are complex syndromes that arise due to external stressors like ischemia, hypertension, or metabolic syndrome. These conditions often involve secondary molecular dysfunctions such as calcium-handling defects, oxidative stress, and maladaptive remodeling—making them suitable for functional gene modulation using AAVs.

The G protein-coupled receptor kinase 2 (GRK2) is upregulated in the failing myocardium and contributes to post-MI sympathetic overactivation. In a porcine model of heart failure following left ventricular (LV) myocardial infarction, AAV6-mediated delivery of βARKct, (a GRK2 inhibitor peptide) improved left ventricular hemodynamics and contractility (25). Retrograde injection of either AAV6.βARKct or control AAV6.luciferase into the anterior inter-ventricular vein 2 weeks post-MI demonstrated robust and long-term expression of βARKct, resulting in beneficial LV remodeling and plasma norepinephrine levels (25).

S100A1, a calcium-binding protein with positive inotropic effects in cardiac contractility, is depleted in failing cardiomyocytes. In post-ischemic porcine models of MI, targeted AAV9.S100A1 delivery restored sarcoplasmic Ca^2+^ in cardiomyocytes and rescued cardiac contractility (39, 40). In mice with type 2 diabetes-induced HFpEF, the retro-orbital injections of AAV9-cBIN1 have rescued cardiac lusitrophy, improved exercise intolerance, and restoration of transverse-tubule microdomains that improves Ca^2+^ recycling (41). Similarly, in a canine model of ischemic cardiomyopathy-induced chronic heart failure, myocardial delivery of low-dose AAV9-cBIN1 led to marked reversal of disease symptoms (42).

These preclinical studies conducted in small and large animals have clearly suggested AAVs as successful gene transferring tools with inferences to their position outcomes during the introduction of correct gene forms. Based on these results, some of these studies were carried out to clinical trials that presented after this section.

*Clinical cases:* the clinical trial led by Dr. Roger J. Hajjar was the first AAV1-based gene therapy targeting SERCA2a in heart failure. Foundational preclinical studies have shown improved cardiac metabolism and function following SERCA2a gene delivery using AAV vectors in rodent models (43). This led to the CUPID Phase 1 trial, which demonstrated feasibility and early efficacy (44). However, the CUPID2 Phase 2b trial did not meet its primary endpoint, with no significant improvement in clinical outcomes compared to placebo, ultimately leading to discontinuation of the program (45). Gene therapy programs for Danon disease, HC, ARVC, and Friedreich's ataxia are utilizing recombinant AAVrh10-based vectors (46). The phase 1 clinical trial (NCT04179643) using AAV2i8 in heart failure patients has shown promising improvements in therapeutic parameters (46). While preclinical results remain encouraging, the translation of these therapies to clinical success has been limited by several challenges discussed in the “Discussion” section. We presented a few most recent clinical trials in tabular form that appeared to be promising during preclinical studies (Table 3).

**Table 3:** Most recent clinical trials with implications of AAV serotypes-based gene therapy programs in cardiac disorders.

**Sr. No**.	**Clinical trial**	**Target gene**	**Serotype**	**Route of delivery**	**Result**	**Disease**	**References**
1.	SERCA-LVAD	SERCA2a	AAV1	Single intracoronary infusion	Phase IIb/Failed	HF	Greenberg et al. (88)
2.	STOP-HF	SDF-1	Plasmid	Endomyocardial	Phase II	IHF	Chung et al. (98)
3.	Ad5.hAC6	Adenylyl cyclase 6	Adenovirus 5	Intracoronary	Phase II	IHF and Non IHF	Hammond et al. (99)
4.	AB-102^$^	Inhibitor 1 (I-1c)^*^	Chimeric capsids	Intracoronary	Phase I	CHF	ClinicalTrials.gov (NCT04179643) (100)
5.	GenePHIT^$^	Inhibitor 1 (I-1c)^*^	Chimeric capsids	Antegrade Intracoronary	Phase II	Non-ICM	Henry et al. (97)

HF, heart failure; IHF, ischemic heart failure; CHF, congestive heart failure; ICM, non-ischemic cardiomyopathy.

^*^Not detailed information available.

^$^Ongoing studies.

*Cardiotropic AAVs and delivery routes:* AAVs are considered promising vectors for gene therapy in heart failure due to their non-pathogenic nature, long-term transgene expression, and ability to target cardiomyocytes (47). Among the 13 naturally occurring serotypes, AAV1, 6, 8, and 9 are known for strong cardiac tropism, particularly when administered intramyocardially (48). Specifically, AAV9 efficiently transduces cardiomyocytes via systemic delivery, a crucial property for translating gene therapy to clinical use without invasive procedures. Studies have shown that AAV9 enables broad myocardial distribution following intravenous or intracoronary injection and has demonstrated superior performance in delivering therapeutic genes such as SERCA2a, cBIN1, MYBPC3, and S100A1 in various models of heart failure and inherited cardiomyopathies (38, 39, 42).

Engineered vectors such as MyoAAV4A and AAVM41 (based on AAV9) have been developed using directed evolution and DNA shuffling. These serotypes show high myocardial selectivity with minimal hepatic transduction when delivered systemically (49–52). Similarly, AAV9 variants like AAV9.45 and AAV9.61 show 20–30 folds lower liver transduction compared to cardiac tissue, further enhancing selective transduction. Combining AAVs with extracellular vesicles (EV) has also led to the development of EV-AAVs, which improve cardiomyocyte transduction, enhance delivery capacity, and resist neutralizing antibodies (53).

*Immune responses to systemic AAV cardiac gene delivery:* systemic delivery of AAV vectors for cardiac gene therapy is known to activate both innate and adaptive immune responses. Even though AAV usually causes less inflammation compared to other viral vectors, giving high doses can still activate the complement system and sometimes lead to serious side effects like thrombotic microangiopathy (TMA) (54). The adaptive immune system can also recognize the viral capsid or even the transgene as foreign material. In such cases, patients may develop T-cell responses against the AAV capsid, which can destroy the transduced heart cells. Also, B-cells can produce neutralizing antibodies which block the virus (54). In fact, many people already have anti-AAV antibodies in their blood (especially against AAV9, seen in 15%−30% of cases), and this can stop the therapy from working in the heart (55). Using immunosuppressive treatments like corticosteroids before or during therapy might help to reduce inflammation and allow the gene to express properly (54).

*Differences in delivery method and vector pharmacokinetics:* the mode of vector delivery plays a crucial role in determining therapeutic success. In preclinical animal studies, direct intramyocardial injections or high-pressure perfusion techniques are often used to ensure the vector penetrates target tissue efficiently. For instance, in a successful study involving canine models using AAV9-cBIN1, multiple direct injections were administered across the left ventricle, resulting in widespread gene delivery (42). In contrast, the CUPID trial employed a single intracoronary infusion, relying on coronary blood flow for vector distribution. In patients with fibrotic or ischemic heart tissue, this method can lead to uneven distribution—some myocardial regions may receive sufficient vector, while others receive little to none. Additionally, in humans, a substantial proportion of systemically delivered AAVs is sequestered by non-target organs such as the liver and spleen, limiting the amount that reaches cardiac tissue (56).

## Novel target molecules in cardiac therapies

Hyperactive sympathetic signaling plays a major role in chronic heart failure, including dilated cardiomyopathy and cardiac arrhythmia. Adrenergic segments of autonomic nervous system regulate myocardial function through the influence of serum catecholamines. Adrenergic receptors (α2-AR, β1-AR, β2-AR, and β3-AR) present on myocardial tissues respond to neurohormones and catecholamines and modulate intra-cardiac hemodynamics and myocardial contractility (57, 58). Among these, α2-AR receptors are known to suppress sympathoadrenal activity, leading adaptive and protective roles in cardiomyocytes (59). In contrast, β1-AR and β2-AR positively modulate cardiac contractility via Gαs-coupled stimulation of adenylyl cyclase and activation of cAMP/protein kinase. Increased expression of β1-AR receptors has been reported in patients with MI (57, 60, 61). We propose that modulating this balance, by enhancing α2-AR activity or reducing β1-AR/β2-AR expression or signaling, may offer new prospects for the treatment of heart-failure and cardiomyopathy (58, 59). Although preclinical studies introducing GRK2 inhibitors in heart failure animals models showed improved cardiac functions, but clinical trial didn't show positive outcomes (62). GRK expression is upregulated in heart-failure and functionally part of the downregulatory mechanism of β-adrenergic receptors (63).

**Figure 3:**
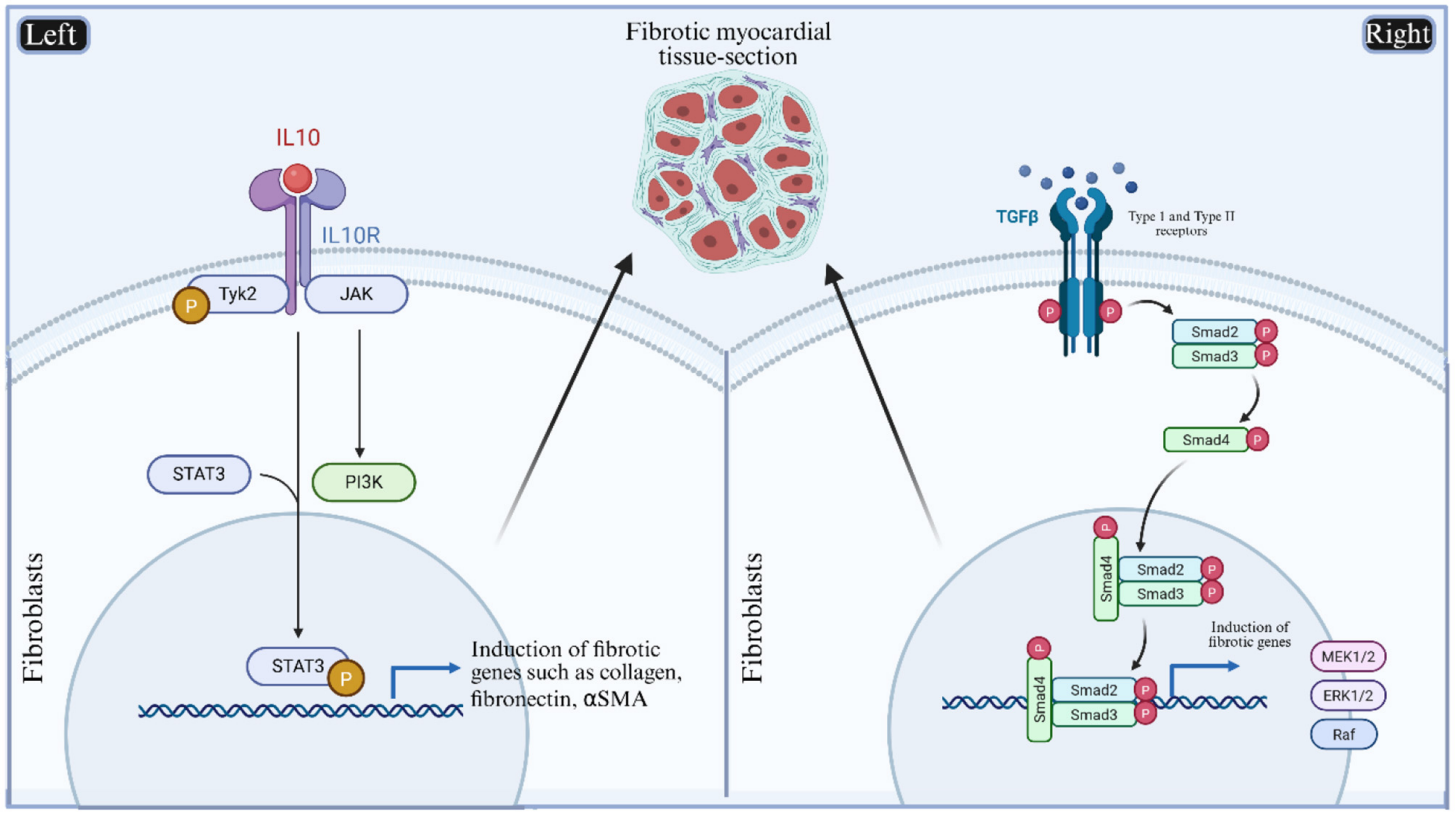
A schematic diagram of signaling cascade of STAT3 and TGF-β molecules within myocardial tissues. STAT3 transcription factor induces programming of initiation of fibrotic genes after getting stimulated with pro-inflammatory cytokines (Left). The TGF-βI and TGF-βII receptors also induce the activation of fibrotic genes in cardiac fibroblasts in Smad2/3 dependent manner (Right). The cardiac fibroblasts have myriads of functions in cardiac pathology (86). Although these pathways have been individually studied in heart disease, their integration into AAV-based delivery frameworks remains underexplored. This figure proposes potential target genes for next-generation cardiac therapy vectors aimed at reversing structural remodeling and chronic dysfunction.

The transcription factor STAT3, an evolutionarily conserved protein, plays various roles in intracellular signaling (64). In cardiomyocytes, STAT3 is known for its protective role via regulating protein kinase A and T-type Ca^2+^ channels. Studies in cardiac-specific conditional *STAT3* knock-out mice have shown pronounced cardiomyocytes hypertrophy, cell death, and subsequent cardiac fibrosis (65). These findings, published in Circulation, suggest the importance of STAT3 in myocardial health. However, we believe these promising results need to be replicated at a larger scale, ideally using large animal models or in human clinical trials. Additional research has shown that STAT3 contributes to myocardial protection by restoring mitochondrial bioenergetics and maintaining calcium homeostasis (66, 67). It has also been implicated in remodeling of the cardiac microenvironment through interactions with cardiac fibroblasts (68). These findings support STAT3 as a promising therapeutic target for ischemia-related cardiac disorders (Figure 3).

Transforming growth factor beta (TGF-β) also plays a complex role in heart failure. Its expression is consistently upregulated in failing myocardium, as reported by several studies (69, 70). Along with experimental studies in animals, patients with dilated cardiomyopathy and hypertrophic cardiomyopathy also reported upregulated expression of myocardial TGF-β (71). All three isoforms of TGF-β are expressed at specific cardiac regions and developmental stages. Mechanistically, TGF-β1 contributes to cardio-protection by suppressing the effects of circulating TNF-α and reactive oxygen species (ROS) in the coronary circulation. Following MI, TGF-β1 expression increases and initiates anti-inflammatory signaling cascades. However, prolonged overactivation of TGF-β1 in pressure-overloaded cardiomyocytes and fibroblasts has been shown to promote cardiac fibrosis and contribute to functional deterioration (72, 73) (Figure 3). Considering the multifaceted role of TGFβ signaling in heart failure, we suggest that TGFβ represents a promising molecular target for the development of next-generation heart failure therapeutics (73).

## Discussion

Heart failure, a devastating endpoint for a wide range of cardiovascular and metabolic disorders, arises from multiple etiologies but converges on a shared pathological pathway. However, the individual risk of progressing to heart failure after an insult varies considerably (74, 75), largely due to genetic variability in myocardial and vascular biology (74, 76).

Therapeutic gene targets for heart-failure have primarily focused on calcium handling and angiogenic pathways. Dysregulated calcium signaling is a hallmark of failing myocardium (77), involving critical components such as sarcoplasmic reticulum Ca^2+^-ATPase, Na-Ca^2+^ exchanger, L-type Ca^2+^ channels, phospholamban, and ryanodine receptors 2 (RyR2) (78). However, despite encouraging data from preclinical and pilot human studies, large clinical trials like CUPID (NCT00534703, NCT01966887) failed to demonstrate therapeutic efficacy and were eventually discontinued (79).

Beyond SERCA2a, other gene candidates such as TRP channels (particularly TRPC3 and TRPC6) have shown relevance in ischemia/reperfusion injury. Genetic knockout or pharmacological inhibition of these channels in animal models has protected cardiac tissue from Ca^2^^+^-induced damage (80, 81). Additionally, over 100 genetic contributors to various forms of cardiomyopathy have been identified (76, 82, 83), and many remain under investigation as potential AAV-based gene therapy targets. Among AAV serotypes, AAV6 and AAV9 showed most efficient tissue-tropism for cardiac-tissues (84) and ability to cross vascular endothelial barriers, and efficacy in both small and large animal models (84). Specifically, AAV9 efficiently transduces cardiomyocytes via systemic delivery, a crucial property for translating gene therapy to clinical use without invasive procedures. Studies have shown that AAV9 enables broad myocardial distribution following intravenous or intracoronary injection and has demonstrated superior performance in delivering therapeutic genes such as SERCA2a, cBIN1, MYBPC3, and S100A1 in various models of heart failure and inherited cardiomyopathies (38–40, 42, 85). AA9′s ability to cross the endothelial barrier (84) and reach deep myocardial layers with high efficiency has made it the leading candidate for cardiac-targeted gene therapy in ongoing and future clinical trials.

Looking ahead, refining gene therapy strategies is essential. Hyperactive sympathetic signaling—common in heart failure and arrhythmias—remains an underutilized target. α2-adrenergic receptor activation and β1-/β2-AR downregulation represents promising therapeutic approaches (58, 59). Similarly, transcription factor STAT3 has emerged as a protective regulator of cardiomyocyte viability, calcium homeostasis, and mitochondrial function (64–68). Although preclinical studies support STAT3 as a therapeutic target, further validation in large-animal models or human trials is needed. Transforming growth factor beta (TGF-β) signaling is another potential avenue, with evidence supporting both protective and pathological roles depending on context (69, 70, 72). Its involvement in fibrosis and immune modulation in the post-infarct heart warrants careful investigation for targeted intervention strategies (73).

To bridge the translational gap between experimental and clinical studies, future strategies must integrate mechanistic understanding of disease pathways with AAV vector design. The clinical cases discussed in this review involve a wide range of pathological mechanisms, including impaired calcium handling, mitochondrial dysfunction, structural protein deficits, and maladaptive remodeling. These diverse pathways highlight the complexity of cardiac diseases and the need for precise therapeutic targeting. Importantly, aligning AAV serotype selection with the underlying molecular pathway—whether it involves sarcoplasmic reticulum calcium cycling (e.g., AAV9 for SERCA2a) (85), cytoskeletal repair (e.g., AAVrh.74 for PKP2) (35), or mitochondrial bioenergetics (e.g., AAV9 for S100A1) (39, 40)—may significantly enhance the efficacy of gene therapy. Future efforts should focus on pathway-stratified AAV engineering and delivery, where serotypes are optimized not only for tissue tropism but also for pathway-specific uptake, expression timing, and transduction efficiency. Such stratification would contribute to a more rational design of gene therapy protocols and expand the knowledge base for precision cardiovascular therapeutics.

## Conclusion

In conclusion, while AAV-based gene therapy continues to hold strong potential for treating cardiac diseases, translational challenges remain. This review highlighted the therapeutic applications of various AAV serotypes, with a particular focus on their efficacy in cardiac gene therapy. Despite encouraging preclinical results, key barriers, such as immune responses, inadequate delivery across the larger human myocardium, and reliance on suboptimal vector designs, have limited clinical success. These challenges, often absent in small animal models, may lead to an overly optimistic assessment of therapeutic benefit during early-phase studies. To address these gaps, future strategies must focus on engineering AAV capsids with reduced immunogenicity and developing methods to transiently suppress or bypass host immune responses, including antibody depletion or T-cell modulation. Improving delivery routes, such as regional perfusion, vector encapsulation, or integration with mechanical circulatory support may also enhance transduction efficiency.
